# Deficits in facial expression recognition in male adolescents with early-onset or adolescence-onset conduct disorder

**DOI:** 10.1111/j.1469-7610.2008.02020.x

**Published:** 2009-05

**Authors:** Graeme Fairchild, Stephanie HM Van Goozen, Andrew J Calder, Sarah J Stollery, Ian M Goodyer

**Affiliations:** 1 Developmental Psychiatry Section, Department of Psychiatry, Cambridge UniversityUK; 2 School of Psychology, Cardiff UniversityUK; 3 MRC Cognition and Brain Sciences UnitCambridge, UK

**Keywords:** Emotion recognition, conduct disorder, antisocial behaviour, psychopathy

## Abstract

**Background:**

We examined whether conduct disorder (CD) is associated with deficits in facial expression recognition and, if so, whether these deficits are specific to the early-onset form of CD, which emerges in childhood. The findings could potentially inform the developmental taxonomic theory of antisocial behaviour, which suggests that early-onset and adolescence-limited forms of CD are subject to different aetiological processes.

**Method:**

Male adolescents with either early-onset CD (*n =*42) or adolescence-onset CD (*n =*39), and controls with no history of serious antisocial behaviour and no current psychiatric disorder (*n =*40) completed tests of facial expression and facial identity recognition. Dependent measures were: (a) correct recognition of facial expressions of anger, disgust, fear, happiness, sadness, and surprise, and (b) the number of correct matches of unfamiliar faces.

**Results:**

Relative to controls, recognition of anger, disgust, and happiness in facial expressions was disproportionately impaired in participants with early-onset CD, whereas recognition of fear was impaired in participants with adolescence-onset CD. Participants with CD who were high in psychopathic traits showed impaired fear, sadness, and surprise recognition relative to those low in psychopathic traits. There were no group differences in facial identity recognition.

**Conclusions:**

Both CD subtypes were associated with impairments in facial recognition, although these were more marked in the early-onset subgroup. Variation in psychopathic traits appeared to exert an additional influence on the recognition of fear, sadness and surprise. Implications of these data for the developmental taxonomic theory of antisocial behaviour are discussed.

Facial expressions of emotion serve important communicatory functions, conveying social cues and reinforcing socially acceptable behaviours (Blair, 2003). Accurate recognition of facial expressions assists in understanding the feelings and intentions of others. Impairments in facial expression recognition have been reported in major depressive disorder (Persad & Polivy, 1993) and schizophrenia (Kohler et al., 2003).

In the present study, we investigated facial expression recognition in adolescents with conduct disorder (CD), a psychiatric diagnosis associated with increased levels of aggression and antisocial behaviour (American Psychiatric Association, 1994). Facial expression recognition may be altered in CD because aggressive individuals show a hostile attributional bias (Crick & Dodge, 1994), which leads them to perceive ambiguous social cues as threatening. Although this hostile attributional bias might be expected to lead to enhanced sensitivity to threatening facial signals, empirical data show that adults with high levels of impulsive aggression (DSM-IV Intermittent Explosive Disorder, IED) are impaired at recognising facial expressions of anger or disgust (Best, Williams, & Coccaro, 2002). These findings have been interpreted as evidence for orbitofrontal cortex dysfunction in IED. Studies of patients with confirmed orbitofrontal cortex damage report impairments in facial expression recognition (Hornak et al., 2003; Hornak, Rolls, & Wade, 1996), with particular deficits seen for anger and disgust (Blair & Cipolotti, 2000). Early orbitofrontal damage may lead to a pattern of childhood-onset impulsive behaviour and aggression that has been termed ‘acquired sociopathy’ (Anderson, Bechara, Damasio, Tranel, & Damasio, 1999). The behavioural parallels between individuals with CD and those with acquired sociopathy suggest that subtle orbitofrontal damage or dysfunction may be present in the former group, which would potentially be reflected in deficits in anger and disgust recognition.

A further issue is whether these deficits are developmentally sensitive in nature, given the distinction made between early- or childhood-onset and adolescence-onset CD in the DSM-IV (American Psychiatric Association, 1994). Moffitt (1993) has suggested that individuals with CD can be divided into those with an early-onset, or ‘life-course persistent’, form of the disorder and those with an ‘adolescence-limited’ form. The early-onset group is proposed to show verbal intelligence quotient (IQ) deficits and associated neurodevelopmental difficulties which play a causal role in the aetiology of their antisocial behaviour. In contrast, the adolescence-limited group is hypothesised to lack significant neuropsychological impairment because their antisocial behaviour arises due to social modelling of deviant peers. Accordingly, one would expect facial expression recognition impairments, if present, to be confined to the *early-onset* subgroup. The study was designed to investigate this question by comparing adolescents with early-onset and adolescence-onset forms of CD, and matched controls.

A related personality variable that may be important in considering the aetiology of antisocial behaviour is variation in psychopathic personality traits. Individuals with CD are on average higher in psychopathic traits than controls (Frick, O’Brien, Wootton, & McBurnett, 1994). A number of studies have demonstrated selective deficits in facial expression recognition in those with high levels of psychopathic traits (Blair, Colledge, Murray, & Mitchell, 2001; Blair et al., 2004; Kosson, Suchy, Mayer, & Libby, 2002; but see Glass & Newman, 2006). Individuals with psychopathic tendencies show impaired recognition of fearful and sad facial expressions (Blair et al., 2001, 2004; Dadds et al., 2006; see Marsh & Blair, 2008, for a meta-analysis of findings in antisocial and psychopathic populations), although one study reported a deficit in disgust recognition in criminal psychopaths when responding with their left hand, but not their right (Kosson et al., 2002).

Blair and colleagues have identified a number of parallels between psychopaths and patients with amygdala lesions (Blair, Peschardt, Budhani, Mitchell, & Pine, 2006). Both groups show deficient acquisition of fear conditioning (Lykken, 1957; Hare & Quinn, 1971; LaBar, LeDoux, Spencer, & Phelps, 1995), impairments in fear recognition (Adolphs, Tranel, Damasio, & Damasio, 1995; Calder et al., 1996; Blair et al., 2001), and reduced potentiation of the startle reflex by negative visual primes (Angrilli et al., 1996; Funayama, Grillon, Davis, & Phelps, 2001; Patrick, Bradley, & Lang, 1993). These similarities have led to the hypothesis that psychopathy is a developmental consequence of early amygdala dysfunction. Supporting this view, a recent functional neuroimaging study showed reduced amygdala activation during fearful face processing in adolescents with disruptive behaviour disorders and high levels of psychopathic traits (Marsh et al., 2008). Counter to this, in a study of adult psychopaths, fusiform gyrus was less activated during fearful face processing, but there were no group differences in amygdala activation (Deeley et al., 2006). To examine the influence of variation in psychopathic traits on facial expression recognition, we used a self-report measure of psychopathic traits, the Youth Psychopathic traits Inventory (Andershed, Kerr, Stattin, & Levander, 2002), to classify participants as high or low in psychopathy.

The present study compared adolescents with early-onset and adolescence-onset forms of CD and age, IQ, and sex-matched controls, in terms of facial expression recognition accuracy, using the Emotion Hexagon task (Calder et al., 1996). Our primary hypothesis was that early-onset CD would be associated with impaired recognition of negatively-valenced expressions, particularly anger and disgust. The possibility of facial expression recognition impairments in those with adolescence-onset CD was an open question, but one of theoretical and clinical significance since the prognosis for this group may also be unfavourable (Moffitt, Caspi, Harrington, & Milne, 2002). We predicted impaired fear and sadness recognition in participants high in both psychopathic traits and antisocial behaviour. The Benton Test of Facial Recognition (Benton, Hamsher, Varney, & Spreen, 1983) was used to assess for possible group differences in facial perception skills, which could impact on the facial expression recognition results. No group differences on this test were anticipated.

## Materials and methods

### Participants

The Local Research Ethics Committee approved the study, and all participants provided written informed consent. The sample consisted of male adolescents aged 14–18 years. Participants were recruited from secondary schools and further education colleges, pupil referral units, and the Cambridge Youth Offending Service. This recruitment process yielded 81 participants with CD, of whom 42 had early-onset CD (EO-CD) and 39 had adolescence-onset CD (AO-CD) or oppositional defiant disorder (ODD; 8 had AO-ODD only). Participants were allocated to the EO-CD group if they or their caregivers reported at least one CD symptom and functional impairment was present prior to the age of 10 years (American Psychiatric Association, 1994), or if they met full criteria for ODD before age 10 and developed CD after the age of 10. If symptom onset occurred after age 10, an AO-CD/ODD diagnosis was given. The majority (77/81) of index cases had a current diagnosis of CD/ODD. We also recruited 40 age- and IQ-matched healthy controls (no history of CD/ODD and no current psychiatric illness).

Exclusion criteria for participation included IQ < 75 as assessed using the Block Design and Vocabulary sub-tests of the Wechsler Abbreviated Scale of Intelligence (Wechsler, 1999), presence of pervasive developmental disorder or chronic physical illness, and use of steroid medication.

Participants were assessed for the presence of CD, ODD, attention deficit hyperactivity disorder (ADHD), major depressive disorder (MDD), generalised anxiety disorder (GAD), obsessive compulsive disorder (OCD) and post-traumatic stress disorder using the Schedule for Affective Disorders and Schizophrenia for School-Age Children-Present and Lifetime version (K-SADS-PL; Kaufman et al., 1997) which reflects DSM-IV criteria (American Psychiatric Association, 1994). Diagnostic interviews were carried out with the participants themselves, and in the majority of cases, their main caregivers.

Psychopathic traits were measured using the Youth Psychopathic traits Inventory (YPI; Andershed et al., 2002), a 50-item self-report measure designed to assess the core affective and interpersonal features of psychopathy. Each item is answered using a 4-point Likert scale ranging from ‘Does not apply at all’ to ‘Applies very well’. The sum score (range: 50–200) is divided by 50 to yield scores ranging between 1 and 4, with higher scores reflecting increased psychopathic traits. Using receiver operating characteristic analysis, Skeem and Cauffman (2003) reported that a threshold of 2.5 out of 4 on the YPI total score provided an optimal balance between the sensitivity and specificity of the YPI in predicting psychopathy as measured using an alternative measure of juvenile psychopathy (the Psychopathy Check List: Youth Version; Forth, Kosson, & Hare, 2003). Thus participants scoring above 2.5 were classified as being high in psychopathic traits. Socioeconomic status was estimated using the reported occupation of the participant’s main caregiver using the Standard Occupational Classification 2000 from the Office for National Statistics (2000).

Ten participants with EO-CD and seven with AO-CD/ODD had current comorbid ADHD. All participants with ADHD had been medication-free for at least 6 months. One AO-CD participant and six EO-CD participants had current comorbid MDD (one EO-CD participant was taking fluoxetine). Four control, seven EO-CD and six AO-CD participants had past MDD. Finally, one AO-CD participant had comorbid GAD and one EO-CD participant had co-morbid OCD.

### Facial identity perception

The Benton Test of Facial Recognition (Benton et al., 1983) assessed participants’ ability to match pictures of unfamiliar faces, to screen for potential deficits in basic face perception skills. The participant has to identify a target face(s) from an array of six faces, presented under different illumination or head orientation conditions. The maximum score is 54 (with scores below 41 indicating impairment).

### Facial expression recognition

**Figure 1:**
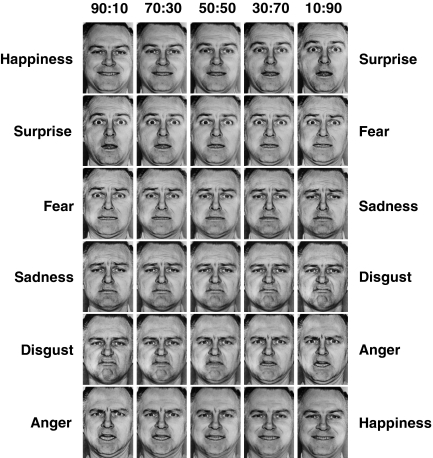
Facial expression continua used in the Emotion Hexagon task. Running from left to right, the columns show 90%:10%, 70%:30%, 50%:50%, 30%:70% and 10%:90% morphs along each continuum. From top to bottom, the continua shown in each row are Happiness–Surprise (top row), Surprise–Fear (second row), Fear–Sadness (third row), Sadness–Disgust (fourth row), Disgust–Anger (fifth row) and Anger–Happiness (bottom row)

Participants completed the Emotion Hexagon task, which was developed to assess accuracy of facial expression recognition (Calder et al., 1996). The task involves judging facial expressions posed by model JJ from the Ekman and Friesen (1975) pictures of facial affect series. These facial expressions are morphed (blended) across continua that span the following six expression pairs: happiness–surprise, surprise–fear, fear–sadness, sadness–disgust, disgust–anger, and anger–happiness (Figure 1). Each continuum consists of five morphed images blended together in the same proportions. For example, images in the happy–surprise continuum contained the following percentages of the happy and surprise expressions: 90% happy – 10% surprise, and then 70% – 30%, 50%–50%, 30%–70%, and 10%–90% of the same two expressions. Happiness is considered correctly rated if the respondent selects ‘happy’ for expressions containing either 90% or 70% happiness. The 50–50% morphs are not scored. The stimulus set contained 30 images in total (6 continua × 5 morphed faces).

The 30 morphed images were presented individually on a computer monitor in random order using PsyScope. Each face was presented for a maximum of 5 seconds and participants were asked to select one of the six expression labels (listed above) that best described the emotional expression. The labels were displayed on the keyboard. Participants were given as long as necessary to make their selection and were not given feedback about their performance accuracy. They completed six blocks, with each block containing one instance of each of the 30 morphed faces. The first block of trials was discarded as practice, which left five blocks available for analysis. In each block, there were two 90% and two 70% expression morphs for each of the six emotions, thus there were four correct instances of each expression. Across the five blocks available for analysis, this gave a total score ranging from 0 to 20 for each expression.

### Data analyses

To investigate possible group differences in demographic characteristics, one-way ANOVA or Chi-Square tests were used. One-way ANOVA was used to compare groups on the Benton Test of Facial Recognition. The Emotion Hexagon data were not normally distributed, and could not be transformed to the normal distribution, so non-parametric Kruskal–Wallis tests were used to investigate group differences in recognition of specific emotions. Where main effects of group were detected, Mann–Whitney U tests were used to perform follow-up pairwise group comparisons. To reduce alpha inflation in these latter analyses, an alpha of .008 (i.e. .05/6) was used. In the comparisons between groups low or high in psychopathic traits, we again used an alpha of .008. Effect sizes are presented as ‘r equivalent’ (Rosenthal & Rubin, 2003) (abbreviated to ‘r’; small ≥ .10, medium ≥ .30, large ≥ .50 (Cohen, 1988)). Analyses were conducted using SPSS 11.5 (SPSS Inc., Chicago, IL).

## Results

### Demographic characteristics

Table 1 shows the demographic characteristics of the sample. The EO-CD group had more lifetime CD symptoms than the AO-CD group. Both CD groups had significantly higher levels of psychopathic traits, as assessed by the YPI, than controls.

**Table 1:** Demographic characteristics

	CON (*n =*40)	AO-CD (*n =*39)	EO-CD (*n =*42)	*p* value	Post-hoc
Age (yrs)	15.8 ± .9	15.5 ± .8	15.8 ± .8	.190	
IQ	96.3 ± 7.7	96.7 ± 11.4	92.5 ± 10.2	.115	
CD symptoms	.4 ± .8	5.1 ± 2.8	8.3 ± 3.1	<.001	CON < AO < EO
MDD diag	0*	1 (2.6%)	5 (11.9%)		
MDD symptoms	.3 ± 1.7	2.5 ± 5.1	3.3 ± 5.4	.007	CON < EO
ADHD diagnosis	0*	7 (17.9%)	10 (23.8%)		
Psychopathic traits (YPI)	2.2 ± .3	2.4 ± .3	2.5 ± .4	<.001	CON < AO, EO
SES					
Low	5 (12.5%)	7 (17.9%)	17 (40.5%)		
Middle	4 (10.0%)	11 (28.2%)	11 (26.2%)		
High	28 (70.0%)	17 (43.6%)	8 (19.0%)		
Ethnicity					
White	37 (92.5%)	32 (82.1%)	41 (97.6%)		
Mixed-race		4 (10.3%)	1 (2.4%)		
Black	2 (5.0%)	1 (2.6%)			
Asian	1 (2.5%)	2 (5.1%)			

*Notes*: Socioeconomic status (SES) information was unavailable for 3 CON, 4 AO-CD, and 6 EO-CD participants. *Presence of MDD or ADHD was an exclusion criterion for the CON group. ADHD = attention-deficit/hyperactivity disorder; AO-CD = adolescence-onset conduct disorder; CON = control; EO-CD = early-onset conduct disorder; IQ = intelligence quotient; MDD = major depressive disorder; YPI = Youth Psychopathic traits Inventory. All data show mean values (± SD) or number (and % of group in parenthesis).

There were fewer non-white participants in the EO-CD group than in the AO-CD group (χ^2^(1)*=*5.14, *p <*.05), but no other group differences in ethnicity were present. Controls were of higher socioeconomic status (SES) than both CD subgroups (*p <*.05), and the AO-CD group was of higher SES than the EO-CD group (*p <*.05).

### Facial identity recognition

There were no significant group differences on the Benton Test of Facial Recognition (*F*(2,118)*=*2.08, *p =*.13). Mean (± SD) scores for the control, AO-CD and EO-CD groups were, respectively, 45.5 (± 3.2), 46.7 (± 3.3), and 45.5 (± 2.6).

### Facial expression recognition

**Figure 2:**
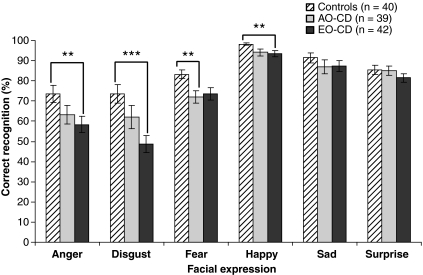
Accuracy of facial expression recognition by experimental group. Relative to controls, anger, disgust and happiness recognition were impaired in the EO-CD group, whereas only fear recognition was impaired in the AO-CD group. **p <.01; ***p <.001. AO-CD: adolescence-onset conduct disorder; EO-CD: early-onset conduct disorder

The Emotion Hexagon results are shown in Figure 2. We first conducted Kruskal–Wallis tests to examine whether there were systematic differences between groups for each expression. There were group differences for anger (χ^2^(2) = 8.9, *p =*.01), fear (χ^2^(2) = 7.5, *p =*.02), disgust (χ^2^(2) = 13.6, *p <*.001) and happiness (χ^2^(2) = 8.1, *p =*.02), but not for surprise or sadness. We subsequently performed pairwise comparisons using Mann–Whitney tests to compare groups for each of these expressions separately.

Relative to controls, AO-CD participants showed deficits in recognition of fear (U = 508.00, Z = –2.69, p = .007, r = .29), but not disgust (U = 663.50, Z = –1.15, *p =*.25) or happiness (U = 659.50, Z = –1.42, *p =*.16). AO-CD participants also showed a marginally significant deficit in anger recognition (U = 584.00, Z = –1.93, *p =*.054, r = .21). Using α = .008, only the fear recognition deficit was significant.

Relative to controls, participants with EO-CD showed deficits in recognition of anger (U = 529.50, Z = –2.89, *p =*.004, r = .31), disgust (U = 423.00, Z = –3.88, *p <*.001, r = .40), fear (U = 630.00, Z = –1.96, *p =*.05, r = .21), and happiness (U = 560.50, Z = –2.92, *p =*.003, r = .31).

The differences for anger, disgust and happiness recognition were significant using α = .008.

The only difference between the AO-CD and EO-CD groups was in recognition of disgust (U = 611.50, Z = –1.97, *p =*.049, r = .22), showing that disgust recognition was impaired in EO-CD participants. This result was not significant using α = .008.

Confusion matrices for each group, showing which emotion labels were selected if the target emotion was misidentified, are provided in Tables 2A–C. It can be seen that if the emotion was misidentified, participants generally selected an easily confusable prototype expression instead (e.g., surprise for fear). Expressions of disgust and anger were frequently confused with each other in this way, a pattern which was most evident in the EO-CD group (Table 2C).

**Table 2:** Confusion matrix data for (a) the control group, (b) the adolescence-onset Conduct disorder group, (c) the early-onset Conduct Disorder group

	Actual expression depicted					
Identified as:	Anger	Disgust	Fear	Happiness	Sadness	Surprise
(a)						
Anger	**73.6**	12.9	0.0	0.3	0.0	0.0
Disgust	18.1	**73.8**	1.1	0.1	4.9	0.1
Fear	3.5	0.4	**82.9**	0.5	2.4	9.9
Happiness	0.4	0.1	0.5	**98.3**	0.6	4.1
Sadness	0.3	12.4	1.1	0.6	**91.4**	0.4
Surprise	4.1	0.5	14.4	0.3	0.8	**85.5**
(b)						
Anger	**63.2**	21.7	1.4	0.5	1.7	0.6
Disgust	26.7	**62.1**	4.1	0.9	5.1	1.5
Fear	4.5	1.4	**72.2**	1.3	3.1	8.1
Happiness	0.5	0.4	1.0	**94.1**	0.8	3.7
Sadness	1.4	13.6	4.2	1.9	86.9	0.9
Surprise	3.7	0.9	17.1	1.3	2.4	**85.1**
(c)						
Anger	**58.3**	33.8	0.8	0.7	1.7	0.6
Disgust	27.4	**48.7**	5.6	0.6	5.5	2.9
Fear	7.0	3.0	**73.6**	0.7	3.8	9.8
Happiness	1.2	0.6	0.6	**93.6**	0.4	3.8
Sadness	1.8	12.5	3.0	2.3	**87.3**	1.6
Surprise	4.3	1.4	16.4	2.1	1.4	**81.4**

The above table show confusion matrices for the control (a), adolescence-onset Conduct Disorder (b) and the early-onset Conduct Disorder (c) groups. In each case, the facial expression depicted is displayed in the columns, and the group's choice of emotion labels in the rows. Percent correct recognition of the relevant emotion is shown in bold.

To rule out the possibility that group findings were driven by comorbid MDD or ADHD, we subsequently excluded participants with these disorders. The results were entirely unchanged following exclusion of participants with comorbid MDD. Dropping 17 participants with comorbid ADHD significantly reduced power to detect differences, thus alpha was set at .05. Kruskal–Wallis tests showed group differences in anger (*p =*.02), disgust (*p =*.002), and happiness recognition (*p =*.04); however, the group effect on fear recognition was only marginally significant (*p =*.07). Relative to controls, EO-CD participants showed deficits in anger (*p =*.01), disgust (*p <*.001), and happiness recognition (*p =*.01), whereas AO-CD participants showed impaired fear recognition (*p =*.02). Thus our findings do not appear to be explained by co-morbid illness in the CD sample.

### Effect of psychopathic traits on facial expression recognition

Since our *a priori* hypothesis was that psychopathy would be associated with specific impairments in fear and sadness recognition, we examined the possibility that the relationship between YPI score and expression recognition would differ in CD cases and controls.

**Figure 3:**
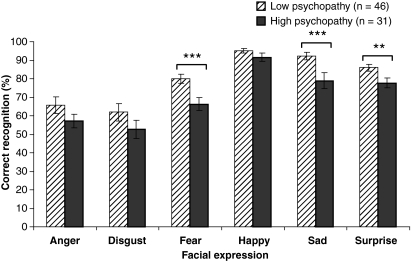
Effect of psychopathic traits on facial expression recognition, when considering participants with CD only (collapsing across the early-onset and adolescence-onset CD groups). Participants with CD who were high in psychopathic traits, as measured using *total* scores on the Youth Psychopathic traits Inventory (YPI), showed specific deficits in recognition of fear, sadness, and surprise relative to CD participants who were low in psychopathic traits. **p <.01; ***p <.001. CD: conduct disorder

We collapsed across CD subgroups and split the sample according to the YPI cut-off of 2.5 (out of 4) for presence of high levels of psychopathic traits. Figure 3 shows accuracy of facial expression recognition in low and high psychopathy CD groups (YPI scores were unavailable for 4 participants).

Comparison of CD participants scoring either low or high in psychopathic traits revealed deficits in recognition of fear (U = 408.50, Z = –3.18, *p =*.001, r = .34), sadness (U = 385.50, Z = –3.50, *p <*.001, r = .37), and surprise (U = 439.50, Z = –2.87, *p =*.004, r = .31) in those high in psychopathic traits. These differences had medium effect sizes, and were all below α = .008. The high and low psychopathic trait groups did not differ in anger (U = 537.50, Z = –1.83, *p =*.07), disgust (U = 588.50, Z = –1.30, *p =*.20), or happiness recognition (U = 582.50, Z = –1.48, *p =*.14).

Repeating these analyses using an aggregate of the callousness, unemotionality and remorselessness YPI subscales (the ‘callous-unemotional dimension’ identified by Andershed et al., 2002) yielded similar, albeit statistically weaker findings. Splitting the CD group along this dimension yielded deficits in fear (*p =*.05) and sadness recognition (*p =*.02) in high callous-unemotional compared to low callous-unemotional trait CD participants (see Supplementary Figure 1). These results were not significant using α = .008.

Differences between the low and high psychopathy groups using the YPI total score were not accounted for by disparities in IQ (mean (± SD): low psychopathy = 95.7 (± 11.2), high psychopathy = 94.1 (± 10.4); *t* (75)<1), Benton performance (*t* (75) = 1.2, *p =*.23), or CD symptoms (mean (± SD): low psychopathy = 6.83 (± 2.89), high psychopathy = 6.58 (± 3.82); *t* (75)<1).

Few control participants scored above the 2.5 cut-off on the YPI (*n =*6). It was therefore not possible to evaluate the influence of variation in psychopathic traits on facial expression recognition in controls.

## Conclusions

The present study had three key aims: (i) to examine whether facial expression recognition deficits are present in adolescents with CD; (ii) to investigate whether these deficits, if present, are developmentally sensitive, i.e., confined to participants with EO-CD; and (iii) to assess the influence of variation in psychopathic traits on facial expression recognition.

Consistent with our hypotheses, the current findings, which relate to a large, well-characterised community-based sample of adolescents with CD, show marked deficits in the recognition of anger and disgust in EO-CD. In addition, the EO-CD group showed impaired recognition of happiness. This may be explained by a ceiling effect in control group performance for happiness recognition. Alternatively, it is worth noting that Dolan and Fullam (2006) reported impaired happiness recognition in personality-disordered offenders, and that happiness recognition is positively correlated with amygdala volume (Kipps, Duggins, McCusker, & Calder, 2007), an area that is reduced in volume in individuals with EO-CD (Sterzer, Stadler, Poustka, & Kleinschmidt, 2007).

The range of deficits was reduced in participants with AO-CD, relative to EO-CD, but impairments were observed in fear recognition compared to controls. No significant differences in facial expression recognition were observed between the EO-CD and AO-CD groups. The expression recognition deficits observed in the CD subgroups were not due to group differences in basic face perception skills, since all three groups showed similar performance levels on the Benton Test of Facial Recognition. Interestingly, we observed a separate influence of psychopathic traits on expression recognition when collapsing across the CD subgroups. Recognition of fear, sadness, and surprise was shown to be disproportionately impaired in participants with both a CD diagnosis and high levels of psychopathic traits, relative to those with CD but low levels of psychopathic traits. This finding was most evident when using YPI total scores rather than callous-unemotional traits.

Our results are not amenable to a level-of-difficulty account, i.e., the argument that expressions of anger and disgust were most difficult to recognise, therefore greatest deficits were seen on those expressions. This view receives further support from our demonstration of differential effects according to level of psychopathic personality traits. Previous research has shown the Emotion Hexagon task to be sensitive to disproportionate impairments in recognition of disgust in patients with Huntington’s Disease (Sprengelmeyer et al., 1996), anger in patients with ventral striatal damage (Calder, Keane, Lawrence, & Manes, 2004), and fear in patients with amygdala lesions (Calder et al., 1996).

The present findings are consistent with previous research showing impaired anger and disgust recognition in patients with disorders of impulsive aggression and putative orbitofrontal cortex dysfunction (Best et al., 2002), and studies of patients with confirmed orbitofrontal lesions (Blair & Cipolotti, 2000; Hornak et al., 1996, 2003). Neuropsychological studies show that the anterior insular cortex is involved in processing disgust stimuli as well as the subjective experience of disgust (Calder, Keane, Manes, Antoun, & Young, 2000; Kipps et al., 2007), thus our findings in EO-CD participants may also be interpreted in terms of insula dysfunction. Consistent with this view, a recent structural magnetic resonance imaging study reported reduced bilateral anterior insular cortex volume in EO-CD (Sterzer et al., 2007). Our demonstration of deficits in anger perception in EO-CD may be considered counterintuitive given extensive evidence for hostile biases in social information processing in individuals with high levels of aggression and/or CD (Crick & Dodge, 1994). However, the present data, together with findings from adults with impulsive aggression (Best et al., 2002), suggest that, despite exhibiting attributional biases in ambiguous social situations, such individuals show reduced, rather than increased, sensitivity to social signals of punishment. Finally, our results are consistent with studies reporting impaired recognition of fearful and sad facial expressions in children with psychopathic tendencies and adult psychopaths (Blair et al., 2001, 2004; Dadds et al., 2006). These findings have been interpreted as evidence for amygdala dysfunction in psychopathy. Of interest, deficient amygdala activation during the processing of fearful expressions has been reported in adolescents with high levels of psychopathic traits and disruptive behaviour disorders (Marsh et al., 2008). The current results are consistent with this view of psychopathy, but additionally demonstrate that individuals with a combination of high levels of antisocial behaviour and psychopathic tendencies exhibit the greatest impairments in facial expression recognition, relative to those with antisocial behaviour only. Most previous studies only compared psychopathic and non-psychopathic individuals (although see Dolan & Fullam, 2006). In contrast, the present study permitted the disaggregation of antisocial behaviour and psychopathic traits, and associated facial expression recognition deficits, apparently showing that these factors contribute in different ways to yield partially dissociable impairments.

### Implications for the developmental taxonomic theory of CD

Whilst our data show that facial expression recognition impairments are most pronounced in EO-CD participants relative to controls, they also demonstrate impaired fear recognition in participants with AO-CD. No differences were observed between the AO-CD and EO-CD groups, apart from a reduction in disgust recognition in EO-CD which did not survive statistical correction. As such, the current findings do not support a strong interpretation of the developmental taxonomic theory, whereby neuropsychological impairments are confined to early-onset CD. This is broadly consistent with previous work showing that other forms of emotion function such as acquisition of differential fear conditioning are equally impaired in both CD subgroups (Fairchild, Van Goozen, Stollery, & Goodyer, 2008).

Three limitations are noted. First, there are difficulties in the interpretation of the Emotion Hexagon results, since the task involves deciding what the dominant emotion is in a particular morph. While this provides a sensitive, quantifiable measure of facial expression recognition ability, forced-choice categorisation of facial expressions lacks ecological validity. In particular, it is not known whether consistently mistaking one emotion for another (e.g. anger for disgust and vice versa) impacts on social communicative ability. Second, classifying age of CD onset with certainty on the basis of retrospective reports is difficult, particularly because the behaviour problems shown by participants with CD may increase during adolescence. Third, eight members of the ‘AO-CD’ group had AO-ODD only. However, it should be noted that CD and ODD are highly interrelated and overlapping disorders (Lahey, Loeber, Quay, Frick, & Grimm, 1992) and ODD is frequently a developmental precursor of CD.

In summary, we demonstrated that both CD subgroups showed impairments in facial expression recognition relative to controls, and although the EO-CD group showed more widespread deficits across multiple emotion categories, there were no significant differences between the CD subgroups. This is consistent with our previous work on autonomic conditioning and suggests that both CD subtypes are associated with impairments in *explicit* and *implicit* measures of emotion processing. Finally, variation in psychopathic traits exerted a separate influence on facial expression recognition in participants with CD.

## Notes

Key points:

- Facial expression recognition deficits are reported in children with psychopathic tendencies, but it is not known whether impairments are seen in those with conduct disorder (CD).
- We demonstrated that both early-onset and adolescence-onset forms of CD are associated with facial expression recognition deficits.
- Qualitatively different impairments were seen in participants with CD and high levels of psychopathic traits compared to those with CD who were low in such traits.
- Both early-onset and adolescence-onset forms of CD are associated with impairments in emotion processing, which may have a neural basis.
- Clinical implications: These impairments may contribute to the interpersonal difficulties observed in CD, and represent potential targets for therapeutic intervention.

## Supporting Information

**Figure 1.** Effect of variation in callous-unemotional traits on facial expression recognition, when considering participants with CD only (collapsing across the early-onset and adolescence-onset CD groups). Participants with CD who were high in callous-unemotional traits, as measured using the ‘callous-unemotional dimension’ of the Youth Psychopathic traits Inventory (YPI), showed deficits in recognition of fear and sadness relative to CD participants who were low in callous-unemotional traits. These differences were not significant using an alpha level of .008. *, p > 05. CD, Conduct Disorder.
