# Supplementary material for: Deficits in facial expression recognition in male adolescents with early-onset or adolescence-onset conduct disorder
Source: J Child Psychol Psychiatry. 2009 May;50(5):627–36. doi: 10.1111/j.1469-7610.2008.02020.x (PMC2737612; doi:10.1111/j.1469-7610.2008.02020.x)
Supplement: Supplementary file 1 [file jcpp0050-0627-SD1.ppt]

## Slide 1
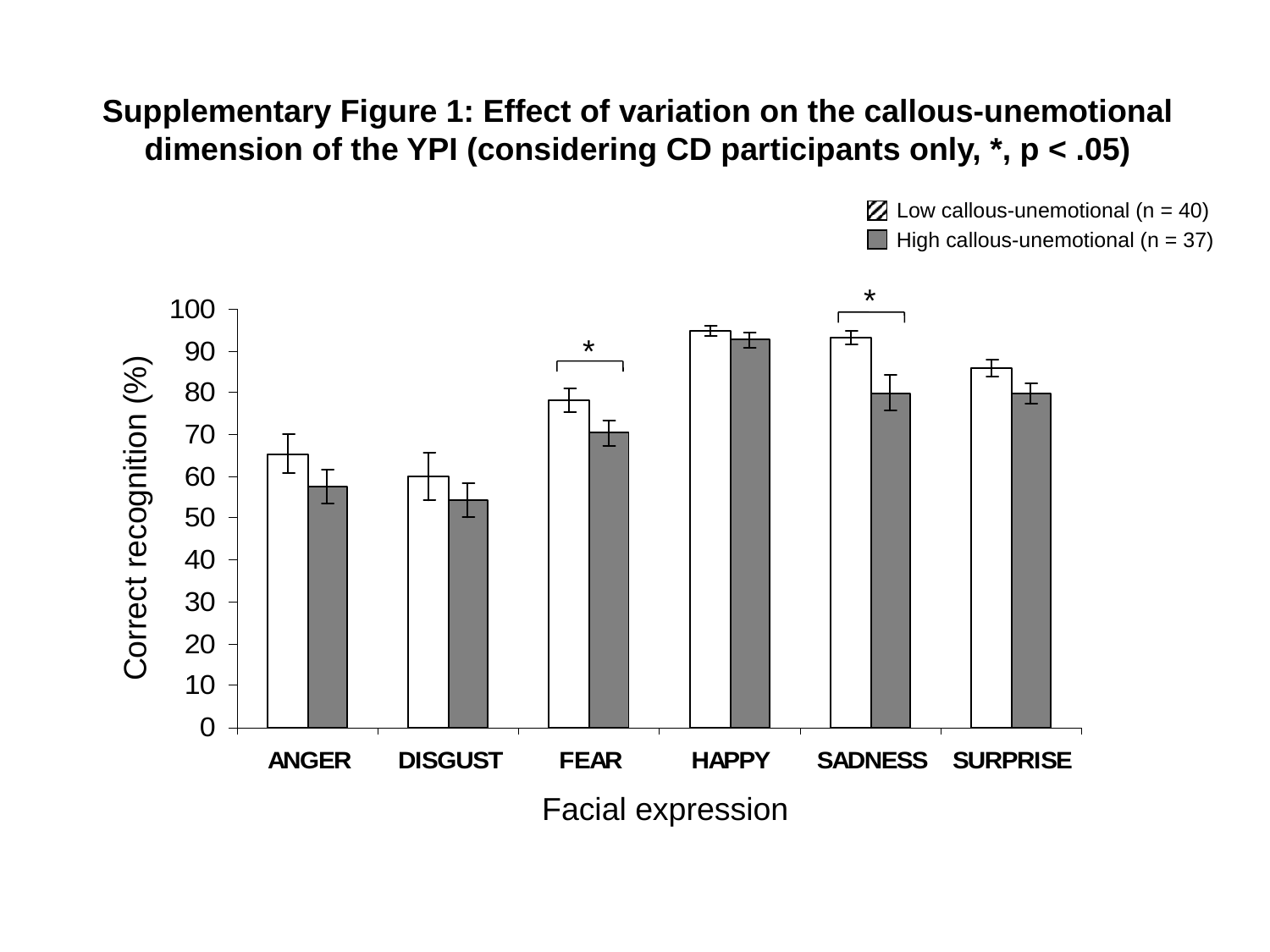

Supplementary Figure 1: Effect of variation on the callous-unemotional
dimension of the YPI (considering CD participants only, *, p < .05)
Low callous-unemotional (n = 40)
High callous-unemotional (n = 37)
*
*
Correct recognition (%)
Facial expression
